# Toxicological and Mutagenic Effects of Particulate Matter from Domestic Activities

**DOI:** 10.3390/toxics11060505

**Published:** 2023-06-03

**Authors:** Daniela Figueiredo, Estela D. Vicente, Ana Vicente, Cátia Gonçalves, Isabel Lopes, Célia A. Alves, Helena Oliveira

**Affiliations:** 1 Department of Biology, Centre for Environmental and Marine Studies, University of Aveiro, 3810-193 Aveiro, Portugal; 2 Department of Environment and Planning, Centre for Environmental and Marine Studies, University of Aveiro, 3810-193 Aveiro, Portugal

**Keywords:** domestic activities, cooking, ironing, cytotoxicity, mutagenicity, PM_10_

## Abstract

People spend most of their time indoors, particularly in their houses where daily activities are carried out, enhancing particulate matter (PM) emissions with consequent adverse health impacts. This study intended to appraise the toxicological and mutagenic responses of particulate matter with a diameter less than 10 μm (PM_10_) released from cooking and ironing activities under different conditions. The cytotoxicity of the PM_10_ total organic extracts was tested in A549 cells using the WST-8 and the lactate dehydrogenase (LDH) assays, while the interference in cell cycle dynamics and reactive oxygen species (ROS) production was analysed by flow cytometry. The *S. typhimurium* TA98 and TA100 Ames tester strains with and without metabolic activation were employed to determine the mutagenic potential of the PM_10_-bound polycyclic aromatic hydrocarbons (PAHs). PM_10_ organic extracts decreased the metabolic activity of A549 cells; however, no effects in the LDH release were observed. An increase in ROS levels was registered only for cells treated with PM_10_ at IC_20_ from steam ironing, in low ventilation conditions, while cell cycle dynamics was only affected by exposure to PM_10_ at IC_20_ from frying horse mackerel and grilling boneless pork strips. No mutagenic effects were observed for all the PM_10_-bound PAHs samples.

## 1. Introduction

It is known that ambient particulate pollution has adverse effects on human welfare [1]; nevertheless, few studies are focused on indoor particles. People spend most of their time indoors, particularly in their dwellings. Exposure to indoor air pollutants can be significant and even more harmful than that experienced in outdoor environments [2,3]. Indoor-produced particles are blamed for up to 30% of illnesses attributable to this pollutant [4]. The health outcomes include mainly lung and respiratory illnesses, but also cardiovascular diseases [4,5]. Besides penetration of outdoor particles, a significant amount of indoor PM is produced while people are cooking [6,7,8], ironing [9,10,11,12], vacuuming [13,14] or heating [15,16,17,18].

Cooking is considered as one of the leading emission sources of particles to the indoor air [8,19,20]. Although more houses in rural areas are using clean energy sources, including electricity and liquefied petroleum gas, many kitchens in developing countries still rely on inefficient forms of combustion in poorly ventilated spaces. These inefficient combustion appliances are fuelled with agricultural residues, charcoal and wood [21,22]. The type of fuel is an important factor for pollutant emissions. Nevertheless, the cooking methods (frying, grilling, stuffing, boiling and roasting), ingredients and oils used, temperature and installed ventilation and exhaust equipment, also contribute to differences in PM emissions [8,23,24]. Thus, the emission profiles cannot be generalised, requiring specific information from different regions.

The organic components, especially PAHs and their derivatives, have been associated with mutagenic and carcinogenic properties [25]. Much of the research undertaken so far, however, has been on outdoor air, and the toxicity of indoor particles has attracted less consideration. Restaurants are an exception, as the chemical composition of cooking oil fumes and their particulate material have been broadly characterised. Some studies were carried out in commercial restaurants [26,27,28,29], whereas others simulated cooking in laboratory kitchens [30,31,32] with a single raw material or activity that does not represent exposure to particles and associated chemical constituents emitted under household conditions. In fact, the cytotoxic and mutagenic effects of PM_10_ emitted from domestic activities, in particular, cooking and ironing, in human cell line models, are scarcely known and, consequently, it is difficult to advise prevention and treatment approaches.

The respiratory tract is the first barrier to interact with inhaled PM, which may deposit deeper into the alveoli, causing irreversible injury to the lungs [33]. The second most prevalent oncological disease and the main determinant of cancer-related death (in both sexes) worldwide is lung cancer [34]. Lung cancer is the fourth most prevalent cancer and the leading cause of cancer-related death in Portugal [35]. Smoking is the main cause of lung cancer; however, only about 10% of smokers are diagnosed with lung cancer and the disease also occurs even when there is no exposure to cigarette smoke [36]. 

It was observed that the cytotoxic potency of the particulate material varies between size fractions and, within a fraction, between locations and sampling periods, indicating that, in addition to size and mass, the chemical composition is the main driver of toxicity [37]. In fact, several researchers have reported as high as or stronger genotoxic, cytotoxic and/or inflammatory effects of coarse particulate matter compared to those of the finest size ranges in different cell lines [38,39,40,41,42,43,44]. Adar et al. [45] conducted a systematic review and meta-analysis of associations for short- and long-term coarse particulate matter (PM_2.5–10_) concentrations with mortality and hospital admissions, reporting increased morbidity and mortality for higher short-term PM_2.5–10_ levels, with stronger relationships for respiratory than cardiovascular endpoints, while these associations were not robust for PM_2.5_. The observations of this epidemiological study are in accordance with previous findings by Brunekreef and Forsberg [46], who concluded that for chronic obstructive pulmonary disease, asthma and respiratory admissions, coarse PM has a stronger or as strong short-term effect as fine PM. Thus, to assess the toxicity or health effects of particulate matter, a good strategy is to consider PM_10_ as it covers both the fine and coarse fractions.

In the present study, an attempt was made to evaluate the potential toxicological and mutagenic effects of PM_10_ in the emissions from cooking and ironing activities in the A549 lung cell line model and the bacterium *Salmonella typhimurium*, respectively. 

## 2. Materials and Methods

### 2.1. PM_10_ Sampling and Chemical Characterisation

The following processes were investigated: cooking different dishes in a residential kitchen and ironing of different types of clothes. The 15.5 m^2^ kitchen was in a single-family home on the periphery of Aveiro, a medium-sized city in Portugal. The kitchen had an electric ceramic hob and a telescopic canopy cooker hood. The experiments were performed with the hood turned on, operating with an air flow at maximum speed of 162 m^3^ h^−1^ and with doors and windows closed. PM_10_ sampling was carried out during the preparation of four dishes: stuffing chicken, frying horse mackerel, and frying and grilling boneless pork strips. Additional details on how the dishes were made can be found in Alves et al [24]. Ironing was carried out in León, Spain, in the living room of a semi-detached house located on the outskirts of the city. Two different types of irons were used: a conventional steam iron and a steam iron with a boiler. The selected garments were composed of various items accumulated by a family of 4 members over the course of a week (jeans, trousers, shirts, t-shirts, blouses, towels, tablecloths, and bedsheets). Measurements took three and a half hours, with no other activities going on in the house. Two ventilation conditions were tested: (i) doors and windows closed (low ventilation) for both irons, and (ii) interior room doors open, and windows closed (normal ventilation) only for the steam iron with a portable boiler. Experimental details can be found in Vicente et al. [47]. 

In the two sets of experiments, a high-volume air sampler equipped with 150 mm quartz fibre filters and running at a flow of 30 m^3^ h^−1^ (details in Tables S1 and S2 of Supplementary Material) was used to collect PM_10_ samples. After gravimetric determination, filter sections were extracted with dichloromethane for 24 h in a heating mantle connected to a distillation condenser, followed by two ultrasonications with methanol, for 10 min each. The extracts were filtered, concentrated, and then evaporated to dryness by N_2_ blow down. The total extracts were split up into different organic classes in a silica gel column applying eluents with gradually increasing polarities. Each organic extract was vacuum concentrated, brought to dryness with nitrogen and characterised by gas chromatography-mass spectrometry. Silylation of polar compounds with OH and COOH groups was carried out prior to chromatographic analysis. Sample extracts and calibration standards were co-injected with internal standards.

### 2.2. Sample Preparation for Toxicological Assays

For cytotoxic assays, PM_10_ filter sections of 47 mm in diameter were extracted with dichloromethane and then two times with methanol, following the procedure described above for the extraction of organics. Total organic extracts were filtered and concentrated in an automated solvent evaporation system to a volume lower than 1 mL. After concentration, samples were dried under nitrogen flow and preserved at −20 °C in glass vials until use. The final extracts were suspended in dimethyl sulfoxide (DMSO).

**Figure 1:**
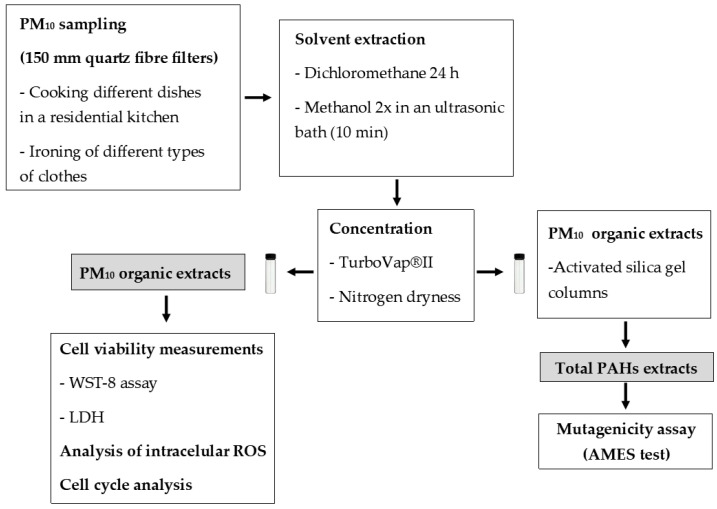
Schematic representation of materials and methods.

For mutagenicity assays, samples were initially extracted as described for cytotoxic assays. Dried extracts were transferred to activated silica gel columns to fractionate the total organic extracts using solvents of distinct polarities. After elution with n-hexane/toluene (8.40:14.10 mL), PM_10_-bound PAHs were dried as described above and suspended into DMSO. A schematic representation of materials and methods is presented in Figure 1.

### 2.3. Cell Culture

The human alveolar adenocarcinoma cell line (A549) was cultured and maintained in Kaighn’s Modification of Ham’s F-12 Medium (F-12K), supplemented with 10% (*v*/*v*) Fetal Bovine Serum (FBS), 1% of Penicillin-streptomycin and 1% Fungizone at 37 °C in a humidified incubator with 5% CO_2_. All experiments were performed when a semiconfluent state was reached (80–90% confluence). To keep cells in optimal culture conditions and actively growing, it is necessary to renew the growth medium and subculture them at regular intervals, usually when 80–90% confluency is reached. In this way, cellular competition for space or lack of nutrients that would lead to cell death are avoided.

### 2.4. Cell Viability Measurements

Cell viability was assessed through the WST-8 assay (CCK-8 Kit) following the manufacturer’s instructions with slight modifications. Briefly, 4 × 10^4^ cells/mL were seeded in 96-well plates and incubated at 5% CO_2_ and 37 °C for 24 h to allow cell adhesion. Cells were treated with eight different concentrations of PM-containing medium (0.1, 0.5, 1, 5, 10, 50, 100 and 150 µg mL^−1^) for 24 h in triplicate. Control groups were treated with the same volume of DMSO and culture medium. Following treatment, 10 µL of WST-8 reagent in fresh culture medium was added to the wells and incubated for 2 h under culture conditions. The absorbance was measured at 450 nm in a microplate reader.

The extracellular LDH was analysed from cell culture supernatants by using a cytotoxicity detection kit in accordance with the manufacturer’s instructions. The A549 cells were seeded and treated with PM_10_ extracts as previously described for the WST-8 assay. After 24 h treatment, 50 µL of cell culture supernatant was removed from each well and transferred to a new 96-well plate. To obtain the highest LDH release, cells were treated with Triton-X 100 (2% in culture medium-positive control) for 10 min at 37 °C, and then treated as the samples. The supernatants were then incubated up to 30 min at room temperature, protected from light, with the reaction mixture. The absorbance was immediately measured at 490 nm. The blank absorbance was measured using cell-free medium under test conditions, and the values obtained were then subtracted from those of the samples. 

### 2.5. Analysis of Intracellular ROS

The levels of intracellular ROS were measured using the fluorescent probe 2′,7′-dichlorofluorescin diacetate (DCFH-DA). A549 cells were seeded in 12-well plates at a density of 4.5 × 10^4^ cells/mL and incubated at culture conditions for 24 h. After 24 h, cells were exposed to PM_10_ at the concentration of the IC_20_, both for cooking and ironing samples, for 24 h in duplicate. After incubation, a set of wells were incubated with 10 mM H_2_O_2_ at 37 °C for 30 min, as positive control. Following a PBS wash, cells were treated with DCFH-DA at a final concentration of 10 μM in culture medium without FBS for 30 min at 37 °C in the dark. The plate was then washed again with PBS, trypsinised, and resuspended in medium with 2% of FBS, and the DCF fluorescence was analysed in Attune^®^ Acoustic Focusing Cytometer within 45 min. Collected data were analysed using the FlowJo software (Table S3).

### 2.6. Cell Cycle Analysis

Cell cycle was evaluated by flow cytometry following a method previously described [48]. Cells were seeded and treated as mentioned above for ROS assay. Cells were collected by trypsinization after cell treatment, centrifuged at 700× *g* for 5 min, and then washed with PBS. Cells were fixed with 85% cold ethanol and kept at −20 °C until analysis. Before analysis, cells were rinsed with PBS and filtered through a nylon mesh into test tubes. Following a 10 min period with 50 µg mL^−1^ ribonuclease A (RNAse), cells were incubated at room temperature for 20 min, in the dark, with 50 µg mL^−1^ propidium iodide (PI, ≥94%). Stained cells were analysed with an Attune^®^ Acoustic Focusing Cytometer and for each sample at least 5000 events were acquired. Collected data were analysed using the FlowJo software (Table S3) to determine the percentages of cells at the G0/G1, S and G2/M phases.

### 2.7. Mutagenicity Assay

The total PAH extracts from each sample were screened for mutagenicity. A short-term bacterial reverse mutation assay, the Ames test, using two *Salmonella typhimurium* strains, the TA98 (to determine frameshift mutations) and the TA100 (to determine base pair substitution mutations) was used [49]. The Ames test was performed with and without S9 metabolic activation (rat liver microsomes) to assess the indirect and direct acting, respectively. The two *S. typhimurium* strains were pre-incubated overnight at 37 °C in nutrient broth. The strains were then treated for 20 min with the PAH extracts (50 µL) in 500 µL of sterile phosphate buffer or 500 µL of freshly prepared S9 mixture, and 100 µL of bacterial culture at 37 °C. Each PAH extract was tested at its maximum concentration since the sample volume available was limited. After incubation, 2 mL of top agar was added and rapidly poured onto the surface of the glucose minimal agar plate (to supply support media) with a trace amount of histidine. A solvent control consisting of 50 µL of DMSO and a negative control containing 50 µL of distilled water were included in each assay to determine spontaneous revertants. The reversion capabilities of each strain were verified using a positive control containing recognised mutagens. For experiments with metabolic activation by the S9 mix, 50 µL of 2-aminoanthracene (10 μg/plate) was used for both strains, whereas for experiments without metabolic activation, 50 µL of sodium azide (10 μg/plate) and 50 µL of 2-nitrofluorene (10 μg/plate) were used for TA100 and TA98, respectively.

After 48 h exposure at 37 °C, the number of revertant colonies was counted. The mutagenicity ratio (MR-sample revertant colonies/negative control revertant colonies) was calculated for each sample. A mutagenic effect is considered to occur when the mutagenicity ratio is higher than 2 [49].

### 2.8. Statistical Analysis

The SPSS software was employed to perform the statistical analysis (Table S3). To assess the normality and homogeneity of variances, the Shapiro–Wilk and Levene’s tests, respectively, were applied first. The cytotoxic results obtained with the WST-8 and LDH assays were compared to the control using the non-parametric Kruskal–Wallis test followed by Dunn’s post hoc test, and Bonferroni correction to the *p*-value. The results obtained in the WST-8 viability assay were taken to determine the IC_20_ values to be used in the ROS and cell cycle tests applying the Statistica software. Cell cycle and ROS results were interpreted using ANOVA followed by Dunnett’s post hoc test. ANOVA was also used to analyse the results of the Ames test followed by the Dunnett’s post hoc test for comparisons between the samples and the negative control. Additionally, the MR above 2 was used as criteria to identify mutagenic effects [49]. All differences were taken as statistically significant for *p* < 0.05.

## 3. Results and Discussion

### 3.1. Cell Viability

**Figure 2:**
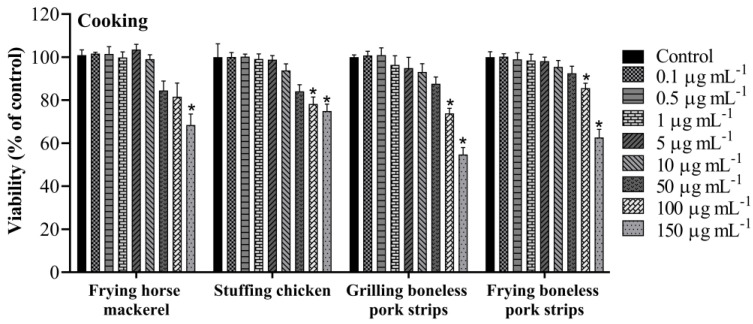
Cell viability assessed through the WST-8 assay after 24 h exposure to PM_10_ collected while cooking. Each bar shows mean ± SD of two independent experiments in triplicate. Asterisks (*) indicate statistical significance compared to control (*p* < 0.05).

**Figure 3:**
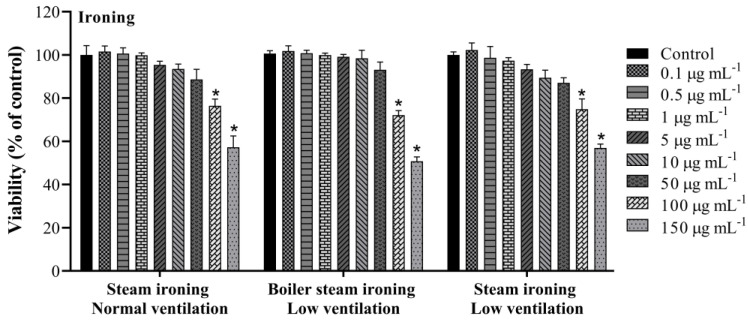
Cell viability assessed through the WST-8 assay after 24 h exposure to PM_10_ collected while ironing. Each bar shows mean ± SD of two independent experiments in triplicate. Asterisks (*) indicate statistical significance compared to control (*p* < 0.05).

The cytotoxic effects were explored by the WST-8 assay after 24 h exposure to PM_10_ organic extracts gathered while cooking and ironing. PM_10_ samples displayed a significant effect on the metabolic activity of A549 cells. As shown in Figure 2, cooking PM_10_ samples had an impact on cells’ viability in a dose-dependent way. PM_10_ emitted while grilling boneless pork strips induced the most cytotoxic effect with a significant reduction to 54.7 ± 3.2% in cell viability. PM_10_ emitted while stuffing chicken, frying boneless pork strips and frying horse mackerel presented a significant reduction to 76.2 ± 0.9, 62.7 ± 3.8 and 71.6 ± 1.7% in cell viability, respectively, at the highest concentration (150 µg mL^−1^). This result is in line with what Alves et al. [24] found, using the *Vibrio fischeri* bioluminescence inhibition bioassay, in which grilling pork emitted “very toxic” particles, while PM_10_ from emissions from the other dishes proved to be less hazardous and was classified as “toxic”. The potential cytotoxic effect of PM_10_ emitted from steam ironing (low and normal ventilation) and from boiler steam ironing (low ventilation) was also measured by the WST-8 assay. The results showed a decrease in cell viability in all studied samples (Figure 3). When compared to the control, samples from steam ironing, under normal and low ventilation, showed a significant decrease in cell viability to 56.4 ± 5.3% and 56.9 ± 1.8%, respectively, at the highest concentration. Boiler steam ironing samples also presented a significant decrease to 50.7 ± 2.0%. As far as we know, the biological effects of PM_10_ emitted while ironing have not been previously described. The cooking results are consistent with earlier research that also showed a reduction in cell responses following exposure of distinct cell lines to PM emissions from cooking. Some studies are focused on the toxic effects of cooking oil fumes (COFs) and Chinese cooking styles and restaurants. Cao et al. [50] explored the effect of COFs in primary ICR mice’s fetal lung type II-like epithelium cells (AECII) for 12 h, 24 h and 48 h using the MTT assay. They found that COFs strongly affect the viability of AECII cells in a dose- and time-dependent manner. At higher concentrations (between 50 and 100 μg mL^−1^), 50% growth inhibition was observed for 48 h treatment. Dou et al. [51] also observed differences in the cytotoxic responses in A549 cells through the MTT test from COFs-PM_2.5_. Treated cells decreased the viability to 86.4, 81.0, and 59.3% in 12.5, 25, and 50 μg mL^−1^ COF particles after 24 h exposure, respectively. Musa et al. [52] studied the biological activities of PM_2.5_ emitted from two simulated cooking experiments and from cooking activities in three Hong Kong restaurants in A549 cells. The authors found a negative dose–response in the viability of the studied cells under 100 and 200 μg mL^−1^ after 24 h exposure. These responses were higher under higher PM_2.5_ concentrations. The researchers reported higher chemical concentrations in a Cantonese food restaurant that used oil-frying methods, suggesting that the cooking conditions and different ingredients could influence the chemical emissions and consequent differences in biological responses. In the present study, a higher decrease in cell viability was observed for PM_10_ emitted from steam ironing, at low ventilation conditions, and from grilling pork strips, indicating that these activities could be more harmful.

### 3.2. Relationship between Chemical Components and Cytotoxic Effects

The complete chemical speciation of PM_10_ has been previously described [24,47]. Correlations between the PM_10_ constituents and the IC_20_ values from the WST-8 viability assay (Table 1) were studied. Negative relationships were found between the cytotoxicity of PM_10_ emitted from ironing activities and the concentrations of phenolic compounds and benzene derivatives, in particular with benzyl alcohol (R^2^ = 0.9522), 5-isopropyl-3-methylphenol (R^2^ = 0.9341), isoeugenol (R^2^ = 0.9286), pyrogallol (R^2^ = 0.9865), 2-methoxy-4-propylphenol (R^2^ = 0.9492) and 4-tert-butylphenol (R^2^ = 0.9889). The PAHs benzo[k]fluoranthene (R^2^ = 0.9413) and benzo[g,h,i]perylene (R^2^ = 0.9846) also displayed a negative relationship with the IC_20_ concentration. Negative relationships were also found for the dicarboxylic acid 1,4-butanedioic (succinic) acid (R^2^ = 0.9318) and the plasticisers benzyl butyl phthalate (R^2^ = 0.9349) and dimethyl phthalate (R^2^ = 0.9899). For cooking emissions, a negative relationship was only observed between the concentration of the benzoic acid alkyl esters (R^2^ = 0.9112) and the IC_20_ values. The obtained relationships suggest that these compounds may be involved in the toxicity of PM_10_ extracts. Negative correlations have also been reported by Musa et al. [52], who performed correlations between cell viability of A549 cells at a concentration of 100 µg mL^−1^ and the concentrations of chemical components. The authors reported negative correlations with the concentrations of benzo[a]anthracene, indeno [1,2,3-cd]pyrene and 1,4-naphthoquinone. Nevertheless, none of these compounds were detected in the present work, which demonstrates the variability of compounds in different types of samples.

### 3.3. Lactate Dehydrogenase Activity

**Figure 4:**
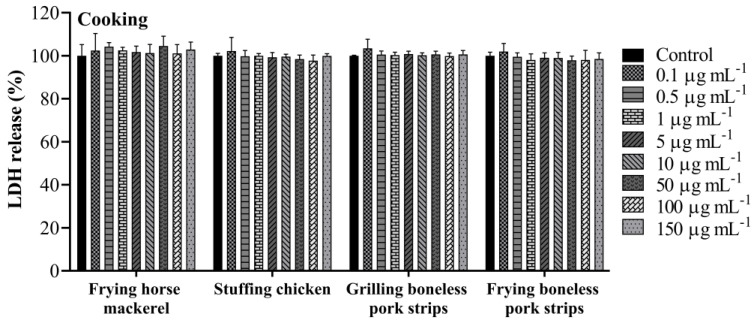
LDH release after 24 h exposure to PM_10_ collected while cooking. Bars represent mean ± SD of two independent experiments in triplicate.

**Figure 5:**
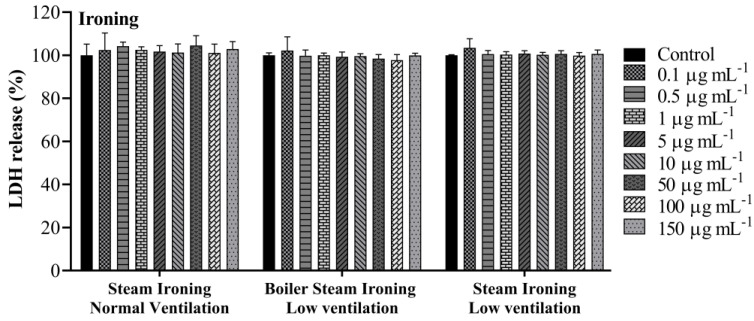
LDH release after 24 h exposure to PM_10_ collected while ironing. Bars represent mean ± SD of two independent experiments in triplicate.

The A549 cellular membrane integrity was evaluated using the LDH test. LDH is released into the cell supernatant when the plasma membrane is compromised, which is a characteristic of cells in apoptosis, necrosis or other types of cell damage. The release of LDH in cell-free supernatant was measured after 24 h exposure to increase concentrations (0.1, 0.5, 1, 5, 10, 50, 100 and 150 µg mL^−1^) of organic extracts of PM_10_ samples collected while cooking and ironing. No effects in the LDH release were observed for the concentrations tested, both for cooking (Figure 4) and ironing (Figure 5) samples. The results of the present study are not in accordance with a previous similar study. Regarding the effects of COFs on alveolar cells, Dou et al. [51] found a significant increase in LDH production when A549 cells were exposed to COF-PM_2.5_ at a concentration of 50 µg mL^−1^ after 24 h treatment. Nevertheless, distinct samples, concentrations and extraction methods were used by Dou et al. [51], which may have led to some discrepancies in the results.

### 3.4. Intracellular ROS

**Figure 6:**
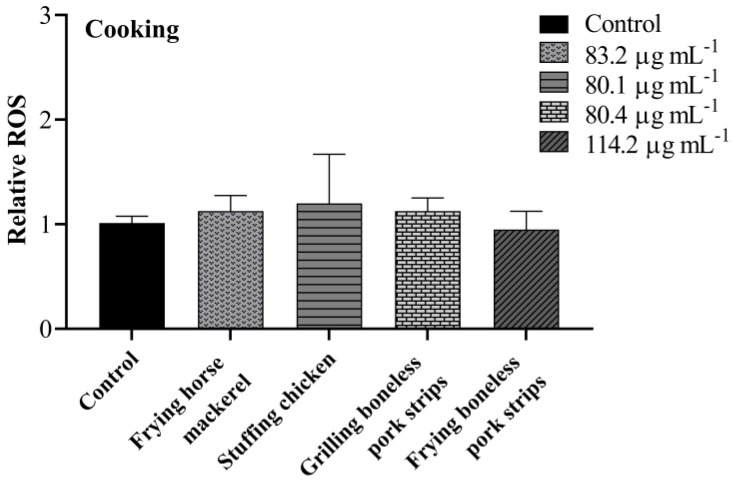
Effects of PM_10_ collected while cooking on production of intracellular ROS after 24 h exposure at the concentration of IC_20_. Bars represent mean ± SD of two independent experiments in duplicate.

**Figure 7:**
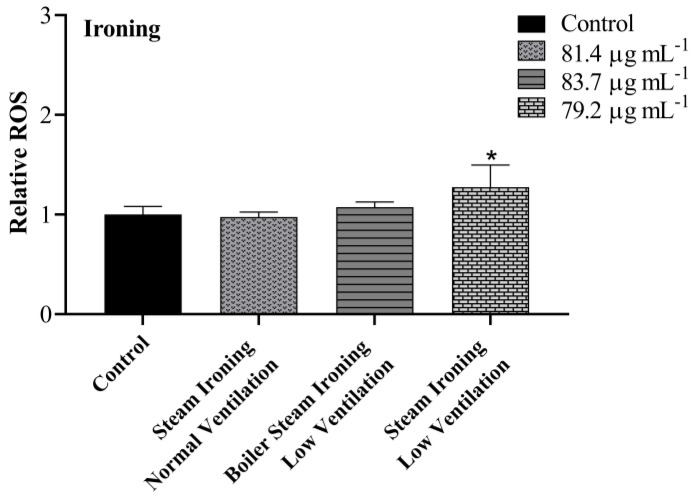
Effects of PM_10_ collected while ironing on the production of intracellular ROS after 24 h exposure at the concentration of IC_20_. Bars represent mean ± SD of two independent experiments in duplicate. Asterisks (*) indicate statistical significance compared to control (*p* < 0.05).

Intracellular ROS production in A549 cells induced by PM_10_ from cooking and ironing activities were evaluated using DCFH-DA and analysed by flow cytometry. As demonstrated in Figure 6, no significant increases in ROS levels were noticed in cells treated with PM_10_ from cooking activities. On the other hand, there was a statistically significant difference in cells treated with PM_10_ from steam ironing, at low ventilation conditions, with an increase of 0.3 in ROS production compared with the control group (Figure 7). A previous study on particulate matter produced from cooking activities in three restaurants in Hong Kong and from two cooking experiments in the laboratory revealed a positive dose–response in ROS generation [52]. The authors found the highest significant ROS generation in cells treated with PM_2.5_ from a Chinese restaurant and from a student canteen at a concentration of 200 µg mL^−1^ after 24 h treatment. Dou et al. [51] also found an increase in intracellular ROS in response to COF-derived PM_2.5_ after 24 h exposure in A549 cells with a significantly increase in ROS at a concentration of 50 µg mL^−1^. In a study performed by Liu et al. [33] on COF-derived PM_2.5_ in primary fetal alveolar type II epithelial cells (AEC II cells), an increase in the ROS levels was also verified. The authors found an increase of 1.14, 1.28, 1.47, and 1.75 times in cells treated with 12.5, 25, 50, and 75 μg mL^−1^ of COF-derived PM_2.5_, respectively. In the present study, no differences in the ROS levels were verified in cooking PM_10_ samples. Different methods of extraction, distinct emission types and sample concentrations may contribute to discrepancies in the obtained results.

### 3.5. Cell Cycle

**Figure 8:**
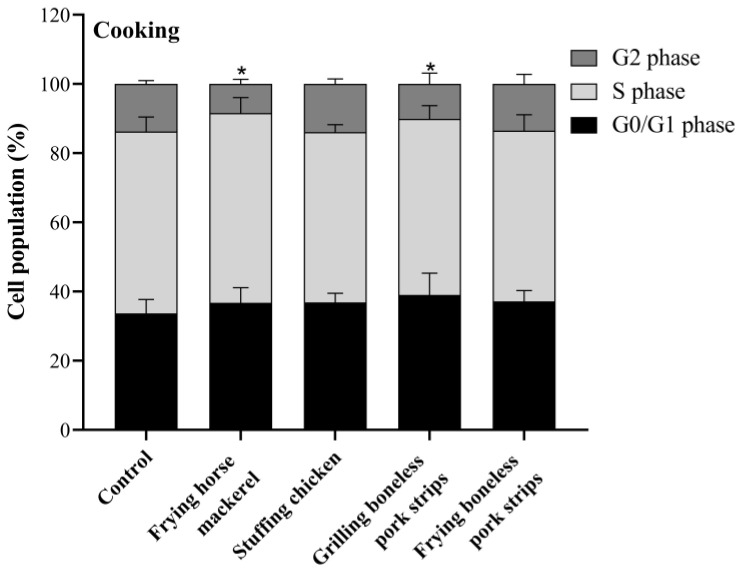
Effects of PM_10_ collected while cooking on cell cycle distribution after 24 h exposure at the concentration of IC_20_. Bars represent mean ± SD of two independent experiments in duplicate. Asterisks (*) indicate statistical significance compared to control (*p* < 0.05).

**Figure 9:**
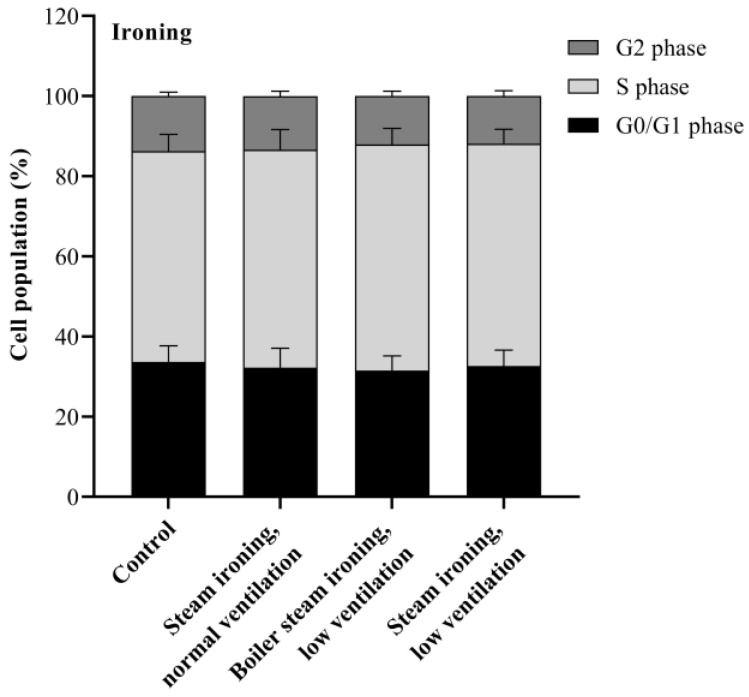
Effects of PM_10_ collected while cooking on cell cycle distribution after 24 h exposure at the concentration of IC_20_. Bars represent mean ± SD of two independent experiments in duplicate.

In an attempt to assess whether PM_10_ interferes with cell cycle progression, cells were exposed to the IC_20_ concentration of PM_10_ from cooking and ironing activities for 24 h. The cell cycle is an important process for cell proliferation, which is controlled by specific molecular mechanisms. The cell cycle comprises checkpoints at each phase that ensure genome integrity. Depending on the type of damage, these checkpoints cause the arrest of cell cycle progression and activate repair processes, or they result in cell death [53]. As can be seen in Figure 8, a significant decrease in the percentage of the A549 cells at G2 phase was found in cells treated with PM_10_ from frying horse mackerel and from grilling boneless pork strips, with a reduction from 14% (control) to 8% and 10% for frying horse mackerel and for grilling boneless pork strips, respectively. It was also possible to verify a slight increase, although not statistically significant, in the G0/G1 phase. This suggests that the mentioned samples inhibit the movement of cells into the G2/M phase. Contrarily, PM_10_ emitted while ironing had no effect on A549 cell cycle dynamics (Figure 9). Regarding the toxicity of COFs in AEC II cells, Liu et al. [33] found that PM_2.5_ treatment caused the arrest of AEC II cells mainly at the G0/G1 phase with a decrease in the percentage of cells in the G2/M phase. Contrarily, Cao et al. [50] observed a significant decrease in the number of cells in the S phase and a significant increase in the number of cells in the G0/G1 and G2/M phases in COFs-exposed cells, which indicates a significant disturbance of these emissions in the normal cell cycle.

### 3.6. Mutagenicity

The organic constituents, especially PAHs and their derivatives, have been associated with potential mutagenic and carcinogenic effects [25]. According to the Ames test results (Table 2), both the TA100 and TA98 strains with and without metabolic activation all displayed mutagenicity ratios below 2—a two-fold principle of mutagenicity confirmation [49]. The sensitivity of the test was demonstrated by the positive controls presenting mutagenicity ratios above 2 with a significant increase (*p* < 0.05) in the number of revertants. Higher ratios were achieved for the TA98 strain, which suggests a tendency for frameshift mutations. Oanh et al. [54] evaluated the mutagenicity of smoke samples, in both gas and PM phases, of three common domestic fuel-stove systems (kerosene, sawdust briquettes, and wood) used for cooking activities also using the TA98 and TA100 stains, with and without metabolic activation. The authors found that the higher mutagenicity emission factor was from wood fuel. They also verified direct mutagenic activities in both PM and gas-phase samples for the TA100 strain (base pair mutations). For the TA98 strain, both direct and indirect mutagenic effects were observed in PM samples of sawdust briquettes and wood fuel; however, in the gas phase, when the S9 was added, a reduction in mutagenicity was registered, indicating a direct frameshift mutagenic effect in the gas phase. No mutagenic effect was verified for samples from the kerosene stove. One important point to know is that lower PAH levels were recorded in our study compared to those reported in other studies. These low PAH levels could be explained by the different food preparation techniques and from the fact that the hood was turned on during all cooking experiments and gas was not used as a fuel.

## 4. Conclusions

The goal of the current study was to evaluate the toxicological and mutagenic potential of PM_10_ obtained in the emissions from cooking and ironing activities. Since cooking is one of the main emission sources of pollutants into indoor air, PM_10_ samples were collected while several dishes were being prepared in a contemporary kitchen with an electric cooktop and an exhaust hood, including frying horse mackerel, stuffing chicken, and grilling and frying boneless pork strips. PM_10_ samples from ironing were also studied under different conditions: a steam iron, a steam iron with a boiler with the room door closed (low ventilation), and a steam iron with the living room door open (normal ventilation).

The metabolic activity of A549 cells was significantly affected by all the particulate sample extracts in a dose-dependent manner, leading to more expressive responses for higher PM_10_ concentrations. These responses were correlated with concentrations of specific chemical constituents in PM_10_, suggesting that the toxicity of PM_10_ emitted while cooking or ironing are influenced by specific chemical species. The most cytotoxic effect was obtained in cells treated with PM_10_ emitted while grilling boneless pork strips. Regarding ironing emissions, PM_10_ from steam ironing at low ventilation conditions proved to have the most cytotoxic effect. Even though, no differences in the LDH release were observed for the concentrations tested, both for cooking and ironing PM_10_ samples. Therefore, the combined results of WST-8 and LDH may suggest that the PM_10_ samples of the present study reduce lung cell viability without affecting cell membrane integrity.

Further experiments were carried out to analyse the potential underlying mechanisms responsible for the observed cytotoxic effects induced by PM_10_ on A549 cells. The results showed that a significant increase in ROS levels was only observed in cells treated with PM_10_ from steam ironing at low ventilation conditions, which may indicate that the increase in the ROS levels could potentiate the decrease in cell viability. On the other hand, alterations in cell cycle dynamics were only observed in cells exposed to PM_10_ from frying horse mackerel and grilling boneless pork strips.

Although several works carried out mainly in restaurants have reported mutagenic effects of cooking-related PAHs, in the present study no mutagenic effects were observed for all tested PM_10_-bound PAH extracts. However, it is necessary to take into account that, in the present study, the small amounts of sample restricted the number of concentrations tested in the Ames assay, so the generalisation of conclusions must be made with caution.

To our knowledge, this is the first study reporting toxicological effects on lung cells from PM_10_ emitted during ironing activities. Although there are several studies about the cytotoxic effects of cooking emissions, discrepancies in the obtained results are observed. This is explained, among other reasons, by the variation in the measured and collected PM emissions, distinct ingredients used and different food preparation techniques, types of fuel used and ventilation conditions.

The current work highlights the significance of understanding the steps leading to lung cancer caused by PM_10_ emitted in indoor residential environments. Increasing the understanding of specific factors in the cause of lung cancer may allow proposing strategies for cancer prevention. The results show that particles released during the preparation of some foods and ironing can trigger biological effects capable of affecting human health, indicating that awareness campaigns are needed to alert people about: (i) the importance of promoting good ventilation while performing domestic activities, (ii) the proper choice of household appliances, namely irons and cooktops, (iii) the need to invest in ingredients that are both healthy and minimise emissions during cooking, and (iv) the option for cleaner energy sources. Additional studies covering other cuisines, indoor domestic environments and activities, and focusing on the toxicological effects of PM emitted indoors are needed to unravel the mechanisms behind PM-induced adverse biological responses.

## Figures and Tables

**Table 1:** Inhibitory concentrations (μg mL^−1^) causing 20% (IC_20_) cytotoxicity on A549 cells with WST-8 assay.

	IC_20_
**Cooking**	
Frying horse mackerel	83.2 ± 8.6
stuffing chicken	80.1 ± 7.0
Grilling boneless pork strips	80.4 ± 4.0
Frying boneless pork strips	114.2 ± 3.1
**Ironing**	
Steam ironing normal ventilation	81.4 ± 5.5
Boiler steam ironing low ventilation	83.7 ± 2.2
Steam ironing low ventilation	79.2 ± 5.6

Values are concentration ± standard error.

**Table 2:** Mutagenicity of PAH extracts of particles collected during cooking and ironing activities to S. typhimurium TA98 and TA100 in the absence (−S9) and presence (+S9) of metabolic activation.

		TA100 −S9		TA100 +S9		TA98 −S9		TA98 +S9	
	ng PAHs/Plate	Revertants/Plate	MR	Revertants/Plate	MR	Revertants/Plate	MR	Revertants/Plate	MR
**Cooking** Fried horse mackerel	7.5	154 ± 30	0.7	149 ± 10	0.89	47 ± 33	1.4	23 ± 3	0.99
Stuffed chicken	4.5	153 ± 13	0.96	142 ± 21	0.85	43 ± 28	1.3	22 ± 4	0.96
Grilled boneless pork strips	7.5	156 ± 12	0.98	158 ± 22	0.94	39 ± 16	1.1	24 ± 3	1.0
Fried boneless pork strips	5	142 ± 19	0.89	184 ± 10	1.1	51 ± 16	1.5	21 ± 4	0.90
**PC**		3663 ± 741 *	23	606 ± 80 *	3.6	121 ± 16 *	3.5	172 ± 28 *	7.4
**DMSO**		159 ± 14		167 ± 5		34 ± 11		23 ± 7	
**Ironing** Steam ironing, low ventilation	11	130 ± 3	0.83	126 ± 8	0.85	13 ± 4	0.74	16 ± 3	0.66
Steam ironing, normal ventilation	10	142 ± 30	0.91	137 ± 10	0.92	14 ± 4	0.82	24 ± 9	1.0
Boiler steam ironing, low ventilation	29	134 ± 22	0.86	143 ± 9	0.96	14 ± 1	0.80	28 ± 2	1.2
**PC**		2598 ± 329 *	17	489 ± 72 *	3.3	129 ± 9 *	7.4	152 ± 14 *	6.5
**DMSO**		155 ± 19		149 ± 7		18 ± 2		24 ± 7	

Values are means ± standard deviation of 3 plates. PC = positive control; MR = mutagenicity ratio. Statistical analysis was performed by one-way ANOVA with Dunnett’s multiple comparison test. Asterisks (*) indicate statistical significance compared to negative control (*p* < 0.05).

## Data Availability

The data presented in this study are available on request from the corresponding author.

## Supplementary Materials

Additional information on equipment, reagents and software can be downloaded at: https://www.mdpi.com/article/10.3390/toxics11060505/s1. Table S1. List of reagents and consumables used in the study; Table S2. Equipment used in the study; Table S3. Software used in the study.
