# Supplementary material for: Toxicological and Mutagenic Effects of Particulate Matter from Domestic Activities
Source: Toxics. 2023 Jun 3;11(6):505. doi: 10.3390/toxics11060505 (PMC10302718; doi:10.3390/toxics11060505)
Supplement: Supplementary file 1 [file toxics-11-00505-s001.zip › toxics-2239011-supplementary.pdf]

Toxicological and mutagenic effects of particulate matter from domestic activities

SUPPLEMENTARY I

Table S1. List of reagents and consumables used in the study.

Name	Brand	Country
Quartz fibre filters	Pallflex®	USA
Dimethyl sulfoxide	Sigma Aldrich	USA
Kaighn's Modification of Ham's F-12 Medium	Gibco, Life Technologies	USA
Fetal Bovine Serum	Gibco, Life Technologies	USA
Penicillin-streptomycin	Gibco, Life Technologies	USA
Fungizone	Gibco, Life Technologies	USA
CCK-8 Kit	Sigma Aldrich	USA
Cytotoxicity detection kit	Roche Diagnostics GmbH	Germany
2′,7′-dichlorofluorescin diacetate	Sigma Aldrich	USA
Ribonuclease A	Sigma Aldrich	USA
Propidium iodide	Sigma Aldrich	USA
Salmonella typhimurium TA100	Trinova Biochem GmbH	Germany
Salmonella typhimurium TA98	Trinova Biochem GmbH	Germany
Rat liver microsomal fractions (S9 fraction)	Sigma Aldrich	USA
2-Aminoanthracene	Sigma Aldrich	USA
Sodium azide	Acros Organics	USA
2-Nitrofluorene	Sigma Aldrich	USA

Table S2. Equipment used in the study.

Equipment name	Brand/Model	Country
High-volume air sampler	MCV, CAV-A/mb	Spain
Automated Solvent Evaporation System	TuboVap II, Biotage	Sweden
Microplate reader	Biotek®	USA
Attune® Acoustic Focusing Cytometer	Applied Biosystems, Thermo Fischer Scientific	USA

Table S3. Software used in the study.

Name	Version	Brand	Country
FlowJo	10	FlowJo LLC	USA
SPSS	26	IBMStatistics	USA
Statistica	10	StatSoft®	USA
Gen5	1.11	Biotek	USA
